# Innovation of new Florescent-epitope technique for immune assay methods

**DOI:** 10.1016/j.mex.2025.103451

**Published:** 2025-06-19

**Authors:** Zahra Farjami, Mohammad Mehdi Akbarin

**Affiliations:** a Department of Modern Sciences and Technologies, Faculty of Medicine, Mashhad University of Medical, Mashhad, Iran; b Biological Sciences Department- Faculty of Higher Education Studies, FESC, UNAM, Mexico City, Mexico; c Islamic Azad University-Mashhad Medical Sciences-Medical School, Iran

**Keywords:** Florescent dye, FAM, BHQ-1, Immunoassay, Epitope-paratope reaction, Prob antibody

## Abstract

The proposed method, which utilizes a FAM fluorescent dye and a BHQ1 quencher complex for indirect analyte assessment through antigen-antibody reactions, represents a significant advancement in immune assay technology. By leveraging the unique physical properties of the FAM-BHQ1 complex, we achieve enhanced accuracy and efficiency in analyte quantification. The complex's fluorescence characteristics, along with its inherent stability and resistance to photobleaching, contribute to improved data reproducibility. Furthermore, the system's adaptability to multiplex assays allows for simultaneous detection of multiple analytes within a single reaction, significantly increasing throughput and reducing assay time. Favorable conditions enhance the method's performance, including a broad dynamic range and minimal background signal. The inherent advantages of this approach—increased sensitivity and specificity, coupled with streamlined workflow—promise to revolutionize immunological research and diagnostics. Implementing this FAM-BHQ1-based method will facilitate more precise and reliable detection and quantification of a broader range of analytes, leading to improved diagnostic accuracy and more effective therapeutic interventions.

• Despite the importance of immune assays, these assays face challenges such as standardization, variability, and issues with sensitivity.
• Our propose is the use of the FAM fluorescent day, which can attach to the Fc region of the antibody and BHQ1
• This limitation is the time and number of epitope-to-paratope attachments.

## Graphical abstract

**Figure:**
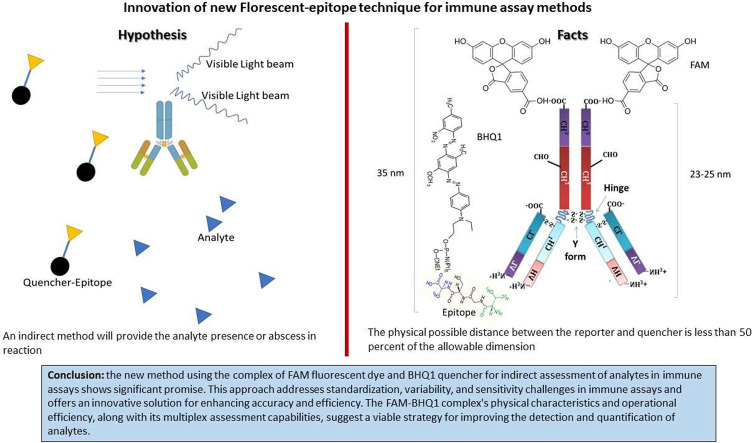

Specifications table

**Table:** 

Subject area:	Immunology and Microbiology
More specific subject area:	Combination of Immune Assay and molecular prob
Name of your method:	Prob antibody
Name and reference of original method:	Immune assay
Resource availability:	None

## Background

In immunology, the immune assay is a fundamental tool for measuring and analyzing immune responses [1,2]. This versatile technique plays a crucial role in understanding the immune system's function, evaluating immune responses to pathogens or antigens, and assessing the effectiveness of vaccines and immunotherapies. Immune assays are based on detecting and quantifying specific immune components, such as antibodies, antigens, cytokines, or immune cells [3,4]. These assays employ various techniques to measure immune responses accurately. Common principles underlying immune assays include antigen-antibody interactions, signal amplification, and detection methods such as enzymatic, fluorescent, or luminescent signals [[5], [6], [7]]. Most methods require a solid phase to separate the bound from the non-bound compartments during reactions. Building on the importance of advanced molecular techniques such as FRET in understanding biomolecular interactions, it is equally crucial to consider the broader landscape of immune response assessment. In this context, several immune assays are utilized in immunological research, each serving a specific purpose and providing valuable insights into immune responses.

Several immune assays are used in immunological research, each serving specific purposes and providing valuable insights into immune responses. Some common types of immune assays include enzyme-linked immunosorbent assay (ELISA), flow cytometry, and immunohistochemistry [[8], [9], [10],^11^]. Each assay has its unique strengths and limitations, making it suitable for different research and diagnostic objectives.

Immune assays have diverse applications in immunological research, clinical diagnostics, drug development, and vaccine testing. In research settings, they study immune responses to infections, autoimmune diseases, cancer, and allergies [1,6,7]. In clinical diagnostics, they aid in diagnosing immune-related disorders, monitoring disease progression, and evaluating treatment responses. Immune assays are also crucial in assessing vaccine efficacy and identifying potential biomarkers for immune-related conditions [12,13].

Immune assays are indispensable tools in immunological research, providing valuable data that drive scientific discoveries and advancements in healthcare. These assays enable researchers to investigate the complex interactions of the immune system, identify disease biomarkers, and develop novel immunotherapies. Immune assays also play a critical role in vaccine development, ensuring the safety and efficacy of vaccines against infectious diseases [[14], [15], [16]]. Förster (Fluorescence) Resonance Energy Transfer (FRET) is a distance-dependent, non-radiative energy transfer between a fluorescent donor and acceptor, typically operating over a 1–10 nm range, with transfer efficiency proportional to the inverse sixth power of the donor-acceptor distance, making it an excellent nanometer-scale “molecular ruler” [17,18]. Successful FRET requires substantial overlap between the donor emission spectrum and the acceptor absorption spectrum, as well as suitable dipole orientation. Recently, innovative FRET‑based strategies have extended its analytical capabilities:

Rahmanian et al. described a dual-mode biosensor for detecting aflatoxin B1, which combines electrochemical and fluorescence measurements. FRET (“on/off”) between a FAM-labeled aptamer and magnetic Fe₃O₄@AuNP@ZIF‑8 constructs enabled ultra-sensitive detection with a reaction volume of ∼50 µL per assay [19].

Chen et al. (2024) reviewed multi-step FRET systems using supramolecular assemblies. These systems achieve energy transfers beyond the 10 nm limit by arranging multiple donors and acceptors via host-guest or coordination scaffolds while still maintaining effective FRET at individual donor-acceptor distances within 10 nm [20].

FAM and BHQ-1 are commonly used in FRET (Fluorescence Resonance Energy Transfer) techniques. FAM acts as the donor fluorophore, and BHQ-1 serves as the acceptor/quencher molecule [21,22]. When FAM and BHQ-1 are nearby, BHQ-1 quenches FAM's fluorescence, reducing or eliminating its emission [23]. This quenching effect is detected and used to quantify the distance between the two molecules, which is a key principle of FRET.

## Method details

**Fig. 1:**
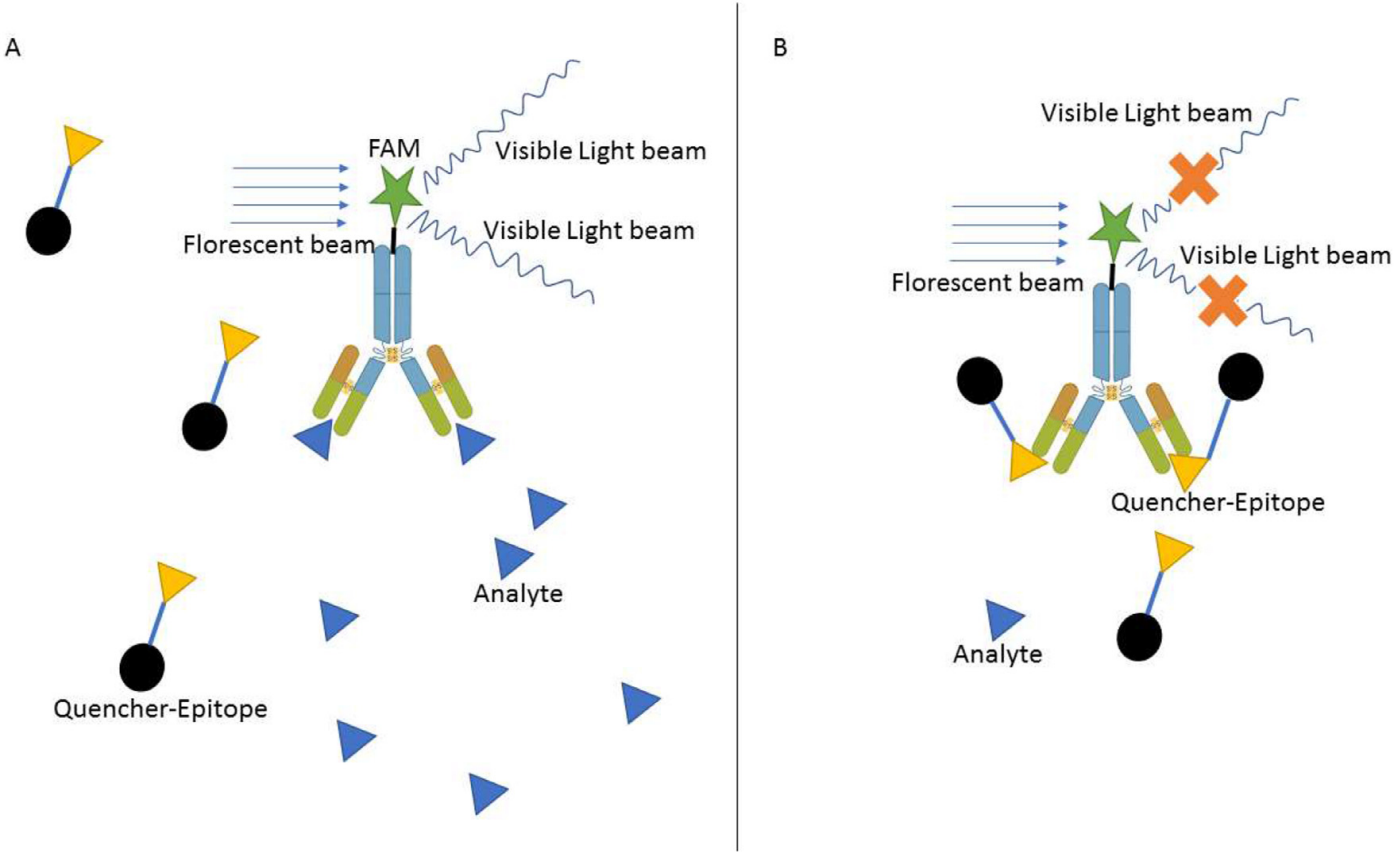
The schematic illustration of the florescent- epitope detection method. **A:** In the presence of target analyte epitopes, it can attach to the specific antibody; therefore, the quencher-linker epitope cannot attach to the FAM antibody to suppress its light production. **B:** In the absence of target analyte epitopes, the BHQ1 as the quencher can attached to the FAM-antibody via a specific epitope to conquer the light emission.

In this study, we propose a novel method for indirectly assessing any analyte that can be detected through an antigen-antibody reaction. Within this proposal, we suggest using the complex of a fluorescent dye and its quencher to demonstrate the attachment of antibodies to their related specific epitopes or antigens. We suggest using the FAM fluorescent dye, which can be attached to the Fc region of the antibody, and BHQ1 as its quencher, attached to the related antigen epitopes. Therefore, if the target analyte is present in the reaction solution, it can bind to specific antibodies. However, after adding the quencher-epitope complex, it cannot bind to the specific paratopes that provide the fluorescent signals in the reaction tube. Furthermore, if the analyte were absent from the reaction, the quencher-epitopes could bind to their specific paratopes and suppress the fluorescent exposure (Fig. 1).

## Method validation

**Fig. 2:**
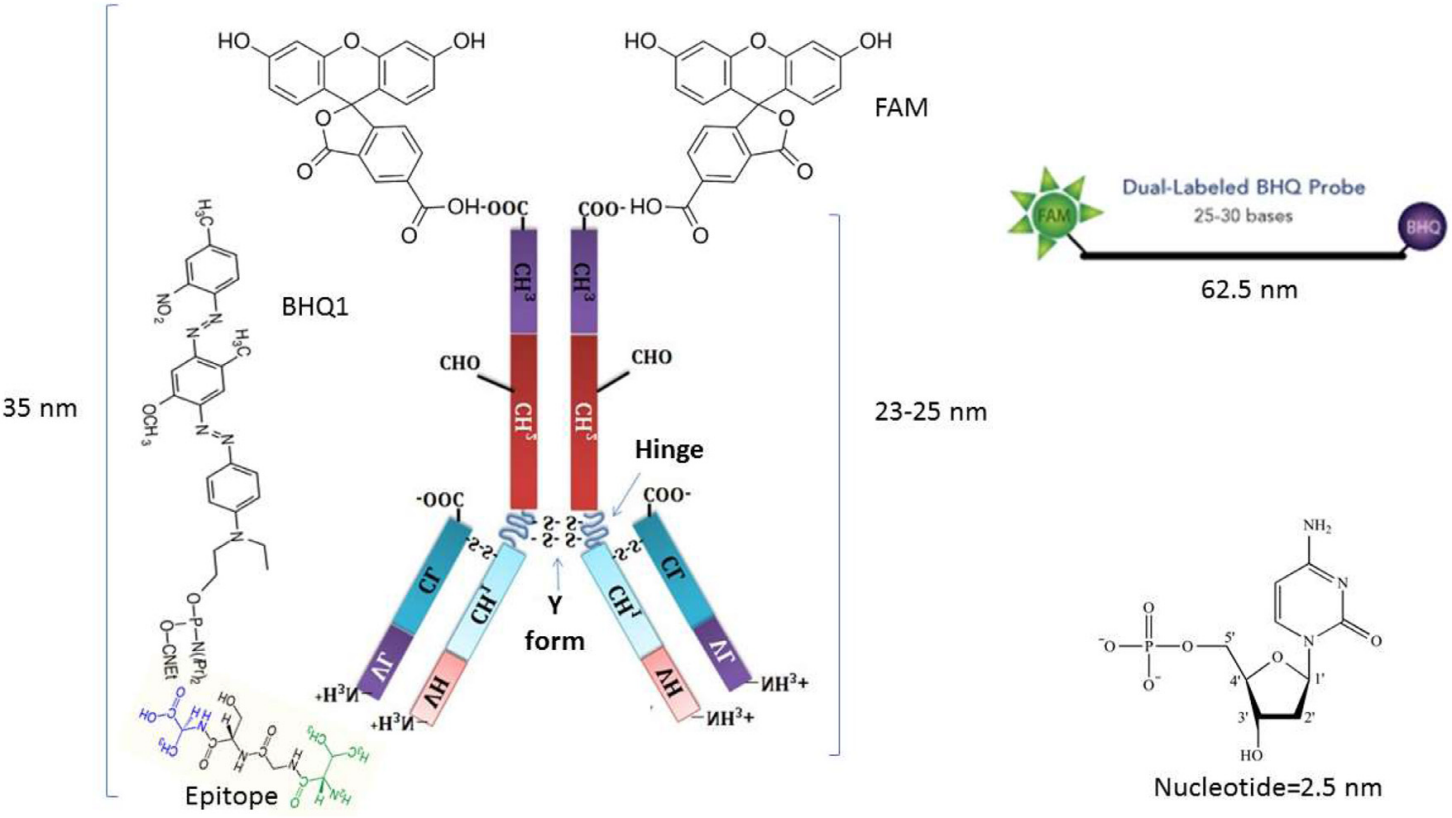
Physical composition of FAM-Ab plus BHQ1-Epitope. The physical possible distance between the reporter and quencher is less than 50 percent of the allowable dimension; therefore, adding a simple linker will not affect the light emission by the complex.

Physical composition: The complex of FAM-BHQ1 is more commonly used in molecular TaqMan Real-time PCR as the productive fluorescent dye in prob. Therefore, the maximum allowable distance between the reporter and quencher complex is 62.5 nm (25 nt × 2.5 nm =62.5 nm) [20,21]. Each antibody, mainly IgG, the most common antibody used in diagnosis, has a physical length of around 10 nm [22,23]. The Y-shaped conformation of Ab led to increased length, which Fab-Fc can increase to 23–25 nm (Fig. 2) [23,24]. In addition, each epitope consists of 5–10 amino acids (aa) by a maximum length of 2–10 nm (one aa =0.4–1 nm, 5–10 aa= 2–10 nm). Therefore, the epitope-paratope complex will provide a maximum prediction of 35 nm.

In addition, in FRET techniques, the key methodological considerations for donor-acceptor distance (R) are calculated as follows:

Efficiency *E*= $\frac{1}{1+(\frac{R}{R0})}$6 with typical Förster radii (Ro) of 3–7 nm (30–70 Å), confirming that effective FRET requires donor-acceptor pairs to be spaced within this range.

Reaction volume:

For small-volume assays, reaction tubes commonly contain 20–100 µL, as demonstrated in Rahmanian et al.'s dual-mode aptasensor [19]. For fluorescence imaging or in-cell FRET, volumes may range higher—100–500 µL—depending on the instrument sensitivity and sample type.

Time of operation: This reaction will be limited to the time of epitope-to-paratope attachment. Every light or signal production incubation time, washing stages, and soaking time will be removed.

Multiplex assessment: By using different reporter quencher compounds, we can evaluate multiple analytes simultaneously in a single reaction. These sets of reporters and quenchers will have different emission wavelengths to avoid spectral overlap.

Increase the limit of detection and decrease reagent consumption: In this method, we can use fewer antibodies than the ELISA Sandwich and indirect methods while maintaining an increased limit of detection. We must use 1 mg/dL of the first and second antibodies in the ELISA method, which can be diluted up to 1:20,000 times. The 1 mg/dL antibody equals 1/15,000 mol, which contains 4.01146 × 10^18 molecules [[25], [26], [27]]. Let us consider the detection limit in this method to be 10^9 molecules. We can obtain at least 1 in 200,000,000 diluted antibodies, and the sensitivity is 10^5 times that of ELISA. Thus, the sensitivity can increase while the price of analysis could be decreased for using the smallest number of antibodies, even if we examine this reaction in the final 10–100 µl volume.

Conditional factors in measurement: The previous study demonstrated that FAM can maintain a constant activity at 40 °C; therefore, the reaction temperature (37 °C) is expected to affect FAM activity [28,29]. Moreover, at pH 7.4, this molecule also exhibits proper signal emission, matching the pH of body fluids, such as serum [29]. The shelf life of FAM is approximately 12 months; therefore, it seems suitable for use in diagnostic reagents [29,30]. Furthermore, the fluorescent dye demonstrated a better limit of detection than colorimetric dyes, such as tetramethylbenzidine (TMB). Additionally, each antibody can simultaneously attach to two FAM-BHQ1 molecules, thereby amplifying the detection signal.

## Limitations

1. The time and the affinity of antibody-antigen interaction.
2. Matrix Interference in Complex Samples:

One of the key challenges in applying fluorescence-based assays—particularly FRET-based detection—in biological samples is the potential for matrix interference, especially from complex biological fluids such as serum or plasma. These matrices contain high concentrations of proteins, lipids, and salts that can affect assay performance in several ways:

2.1. Nonspecific Protein Binding: Serum proteins, such as albumin and immunoglobulins, can adsorb onto assay surfaces or bind nonspecifically to assay components, including fluorophore-labeled probes. This can lead to elevated background fluorescence, reduced specificity, or false-positive FRET signals. To address this potential problem, the sample can be diluted to an allowable scale, considering the limit of detection. In addition, the use of serum blank can be applied to bypass the possible nonspecific bound of serum materials.
2.2. Autofluorescence: Components of serum, including NADH, flavins, and porphyrins, can exhibit intrinsic fluorescence, contributing to background noise and interfering with accurate detection, particularly in the UV to visible range. In these cases, the interfering amount of multifluorescent biomolecules should be identified and mentioned as the possible source of interfering results. By using a serum fluorescent blank, it can be mostly removed.
2.3. Quenching Effects: High ionic strength or the presence of quenching agents, such as bilirubin and hemoglobin, can attenuate the fluorescence signal through static or dynamic quenching, thereby reducing the assay's sensitivity [24,25]. To pass this problem, only samples that are not icteric or hemolytic are valid.

3. Photobleaching possibility:

Despite the widespread use of FAM in fluorescent assays, FAM is particularly susceptible to photobleaching, a process in which prolonged light exposure leads to the irreversible degradation of the fluorophore, resulting in a diminished signal intensity over time [26].

Photobleaching can significantly impact the accuracy and reproducibility of fluorescence measurements, particularly in long-term assays, repeated scans, or when high-intensity excitation is employed. To mitigate this risk:

3.1. Minimize exposure time: Use shorter data acquisition periods and limit laser or lamp exposure during image capture or fluorescence measurement.
3.2. Apply anti-fade/anti-photobleaching reagents: Commercially available reagents, such as ProLong, SlowFade, or Vectashield, can preserve fluorescence by scavenging reactive oxygen species and stabilizing the dye environment.
3.3. Use light-protective handling: Shield samples from ambient light during preparation and storage, and use amber tubes or foil wrapping where applicable.
3.4. Optimize instrument settings: Lowering excitation intensity and increasing detector sensitivity can reduce photobleaching without compromising signal detection.

In studies where fluorescence signal stability is crucial, implementing these strategies helps preserve the integrity of FAM fluorescence and ensures consistent data output throughout the assay.

## Ethics statements

None

Despite their importance, immune assays face issues with standardization, variability, and sensitivity [14,27]. Addressing these challenges is crucial to ensure the reliability and reproducibility of immune assay results. Future advancements in technology, such as high-throughput screening methods, multiplex assays, and artificial intelligence, promise to enhance the efficiency and accuracy of immune assays in immunological research. Moreover, in most detection methods, a solid separation phase is crucial for screening the antigen and antibody-bound complex from non-bound compounds [11,28]. This solid phase should be provided by a microplate, beads, or artificial columns, which leads to more steps in the detection procedure and increases the total detection time (TOD) [11,29] . Therefore, suggesting and designing a new method that summarizes the detection procedure in a single step improves the diagnostic values. Furthermore, In the last two years, fluorescent probes have witnessed significant technological breakthroughs aimed at enhancing sensitivity, temporal resolution, and multiplexed detection:

**Plasmonic nanoantenna-enhanced brightness:** Tiwari et al employed optical nanoantennas to improve single-molecule fluorescence brightness to ∼2 million photons · s⁻¹ · molecule⁻¹, achieving microsecond temporal resolution for studying fast molecular dynamics [30].

**FRETfluor barcoding for multiplexing:** Chu et al. developed over 27 spectrally distinct FRETfluor constructs by tuning donor-acceptor configurations and dye environments, enabling simultaneous single-molecule identification with high accuracy [31]

**Far-red squaraine FRET probes**: Gupta et al. introduced a squaraine-based homo-FRET probe (SQ-122 PC) in 2024 with far-red emission (∼650–700 nm), 93 % quenching efficiency, and femtomolar sensitivity—ideal for deep-tissue enzyme assays [32]

**Two-photon anthocyanidin probes**: Zhang et al. designed anthocyanidin-based two-photon probes with emission beyond 600 nm, significant Stokes shifts (>100 nm), and high two-photon absorption (∼957 GM), enabling deep imaging with minimal background [33].

These advances—ranging from improved brightness and time resolution to spectral multiplexing, deep-tissue span, and reactive probe specificity—significantly enhance the applicability of fluorescence techniques in complex biological settings. They also underscore the evolving landscape of fluorescent methodologies, supporting the relevance of your method within current state-of-the-art imaging and sensing platforms.

## CRediT author statement

None

## Funding

This Study did not received any type of financial support from the organisation.

## Declaration of competing interest

The authors declare that they have no known competing financial interests or personal relationships that could have appeared to influence the work reported in this paper.

## Data Availability

No data was used for the research described in the article.
